# Evaluation of the Anxiolytic Effect of Ramelteon in Various Rat Models of Anxiety

**DOI:** 10.7759/cureus.38717

**Published:** 2023-05-08

**Authors:** KR Nachiappan, Balakrishnan Sadasivam, Ahmad Najmi, Chandrahasan K

**Affiliations:** 1 Pharmacology, Employees' State Insurance Corporation (ESIC) Medical College & Post Graduate Institute of Medical Science and Research (PGIMSR) Model Hospital, Bangalore, IND; 2 Pharmacology, All India Institute of Medical Sciences (AIIMS) Bhopal, Bhopal, IND; 3 Pharmacology, Andaman and Nicobar Institute of Medical Sciences, Port Blair, IND

**Keywords:** neuropharmacology, animal models, melatonin, anxiety, ramelteon

## Abstract

Background

Melatonin was found to have anxiolytic properties in several animal and human studies. The melatonin receptor agonist ramelteon might also have a similar anxiolytic action.

Objectives

The objective of the study was to evaluate the effect of ramelteon in various rat models of anxiety and to explore the possible mechanism of action.

Methods

The anxiolytic effect was compared between the control group, diazepam group (1 mg/kg and 0.5 mg/kg), and ramelteon group (0.25 mg/kg, 0.5 mg/kg, and 1 mg/kg) by an elevated plus maze, light-dark box, hole board apparatus, and open field test in Sprague Dawley rats. Antagonists flumazenil, picrotoxin, and Luzindole were used to explore the possible mechanism of action of ramelteon if it showed an anxiolytic property.

Results

Ramelteon as a standalone drug did not show an anxiolytic effect. However, a combination of ramelteon (1 mg/kg) and diazepam (0.5 mg/kg) showed an anxiolytic effect.

Conclusion

Further studies can evaluate the use of a fixed-dose combination of ramelteon and already-approved anxiolytic drugs to reduce the dose of the latter.

## Introduction

Anxiety disorders present with subjective symptoms like worry and fear accompanied by physical symptoms like palpitations, sweating, and tremors [1]. Across the world, the prevalence of anxiety disorders is almost 28%. Anxiety occurs more often in females than males, and it begins in early childhood or adolescence [2].

The pharmacotherapy of anxiety disorders includes benzodiazepines (BZD), 5Hydroxy tryptophan 1A (5HT1A) agonists, selective serotonin reuptake inhibitors (SSRI), selective noradrenaline reuptake inhibitors (SNRI), and tricyclic antidepressants [1]. The use of these existing drugs is limited by unwanted side effects such as nausea, dependence, cognitive problems, and sexual dysfunction. There are also nonpharmacological treatments like lifestyle changes, physical exercise, and cognitive behavioral therapy. However, all patients do not respond to these interventions. Combined treatment with drugs and behavioral therapy is not superior as compared to drugs alone [3,4]. So, there is a need to explore novel pharmacological agents for treating anxiety. Among the new approaches, the alteration of the melatonergic system has gained significant attention. Although melatonin is primarily involved in regulating circadian rhythm and the sleep-wake cycle, it is also found to have several other pharmacological effects. Some of them are antioxidant, antidepressant, anxiolytic, analgesic, and anticonvulsant activities [5]. Melatonin receptor agonist ramelteon may also have similar properties in addition to its sleep-promoting ability. In this study, the anxiolytic property of ramelteon will be evaluated in rats using experimental models of anxiety.

Melatonin is a hormone secreted from the pineal gland. Melatonin secretion is found to peak at night and in the dark. Melatonin exerts its action through melatonin receptors MT1, MT2, and MT3. Melatonin’s sedative effects are mediated through the melatonin receptors MT1 and MT2. Ramelteon is a selective melatonin receptor - an MT1 and MT2 agonist. The FDA approved ramelteon in 2005 for the treatment of insomnia characterized by difficulty in sleep onset [6]. Now it is most commonly prescribed for delayed sleep phase syndrome [7-9].

Before clinical trials, ramelteon’s sleep-promoting effects were tested on rats, cats, and monkeys [10,11]. Following that, numerous clinical trials in humans were also done. These studies proved the ability of ramelteon to decrease the latency of sleep onset and increase the total duration of sleep. Ramelteon does not change sleep architecture and it does not interfere with rapid eye movement (REM) or non-REM sleep [12].

Ramelteon has demonstrated sleep-promoting effects in both preclinical studies and clinical trials. It is the only sleep-promoting drug approved for insomnia and circadian rhythm disorders without any direct sedating effect and abuse liability [6,12]. Ramelteon promotes sleep phase shifts and decreases inflammatory cytokine levels and neurotrophin levels in patients with co-morbid insomnia and depression [13]. Ramelteon treatment has also shown positive results in treating patients with comorbid insomnia and depression [13]. A 12-week open-label study (Gross et al.) suggests that ramelteon is effective in the treatment of insomnia symptoms for adults with generalized anxiety disorder (GAD) [14]. Chronic administration of ramelteon also improved Post-traumatic stress disorder (PTSD) like behavior in mice [15,16]. A recent study has proved that melatonin has the ability to reduce total sleep deprivation-induced anxiety-like behavior [17]. Melatonin congener ramelteon may also have similar properties, which were investigated in this study. The primary objective of the study was to evaluate the effect of ramelteon in the experimental models of anxiety in Sprague Dawley rats by an elevated plus maze (EPM) test, open field test (OFT), light-dark box (LDB) test, and hole board test (HBT). The secondary objective was to explore the possible mechanism of action of ramelteon in rat models of anxiety, using suitable antagonists.

## Materials and methods

The study was conducted after obtaining approval from the Institutional Animal Ethics Committee (IAEC), AIIMS Bhopal (IAEC approval number-AIIMS/BPL/IAEC/2020/025). The animals were used from the Central Animal Facility of AIIMS Bhopal and all the procedures were conducted inside the same facility. Sprague Dawley rats (N = 72) were obtained and used in this study. The animals were maintained under standard laboratory conditions with the natural dark and light cycle. They were allowed free access to the standard dry rat diet and tap water ad libitum. All rats were allowed to acclimatize for one week before starting the experiments. Eight groups of six rats (n=6) each were used in the initial study: control group (A), diazepam 1 mg/kg group (B), diazepam 0.5 mg/kg group (C), ramelteon 0.25 mg/kg group (D), ramelteon 0.5 mg/kg (E) group, ramelteon 1 mg/kg (F), and combination groups (G, H, and I). Animals were randomized in each group, taking into account different variables like age, gender, and weight. Eight to 12 weeks were acquired for the study. Most of the groups in the study had an equal number of male and female rats, as it is important to consider gender differences in psychiatric behaviors [18]. Randomization was also done to get an equal distribution of different weights in all the groups.

Rats in the diazepam 1 mg/kg group and 0.5 mg/kg were given oral diazepam 1 mg/kg and 0.5 mg/kg, respectively. The diazepam dose was selected on the basis of previously published studies. Rats in the D, E, and F groups received oral ramelteon 0.25 mg/kg, 0.5 mg/kg, and 1 mg/kg, respectively. Rats in groups G, H, and I received a combination of diazepam 0.5 mg/kg and ramelteon. The dose of ramelteon in groups G, H, and I were 0.25 mg/kg, 0.5 mg/kg, and 1 mg/kg, respectively. Dimethyl sulfoxide (DMSO) was used to dissolve both ramelteon and diazepam while given orally.

In both acute and chronic studies, the immobilization stress method was used to induce anxiety in rats [19-21]. Immediately after administering the drug, rats were subjected to immobilization for a period of 90 minutes. Rats were immobilized using a restrainer with head and tail out, which could move freely. In the chronic study, rats were subjected to this 90-minute immobilization stress every day for 14 days. After 90 minutes of immobilization, four pharmacologically validated experimental models - elevated plus maze (EPM), light-dark box (LDB), open field test (OFT), and hole board test (HBT) - were employed to evaluate anxiety. Each animal was tested initially in the EPM and then in the LDB, followed by the OFT and HBT in a single setting.

The second phase of the study was done in four groups of six rats each to evaluate the possible mechanism of action of ramelteon. The maximum effective dose of ramelteon for anxiety was used in this study. Thirty minutes prior to the administration of the particular dose of ramelteon, rats were treated with one of the receptor antagonists. The antagonists used were picrotoxin 1 mg/kg (intraperitoneal or i.p.) flumazenil 1 mg/kg (i.p.). and Luzindole 2.5 mg/kg (i.p). The dose and pretreatment period of all antagonists were decided based on previous studies [20]. Picrotoxin (γ-aminobutyric acid (GABA) - a receptor antagonist) and flumazenil (benzodiazepine receptor antagonist) were used to evaluate the role of the GABAergic system in the postulated anxiolytic action of ramelteon. Luzindole (melatonin receptor antagonist) was used to evaluate the role of the melatonergic system in the postulated anxiolytic action of ramelteon. These antagonists did not affect the anxiety levels and locomotor activity of rats at the above doses [20]. Anxiety was evaluated in the four models 30 minutes after administering ramelteon.

Data analysis

Data were analyzed using the statistical software IBM SPSS Statistics for Windows, version 25 (IBM Corp., Armonk, NY). The null hypothesis was that ramelteon does not show better anxiolytic properties compared to diazepam. The significant difference in outcomes in intergroup data and control groups was calculated using a one-way analysis of variance (ANOVA). Post-hoc analysis of study data was done using the two-tailed Dunnet’s test to compare the different groups with the control group. The Tukey post hoc test was used to analyze inter-group data. Probability values less than 0.05 (p-value < 0.05) were considered statistically significant.

## Results

Elevated plus maze

Acute Study

**Figure 1:**
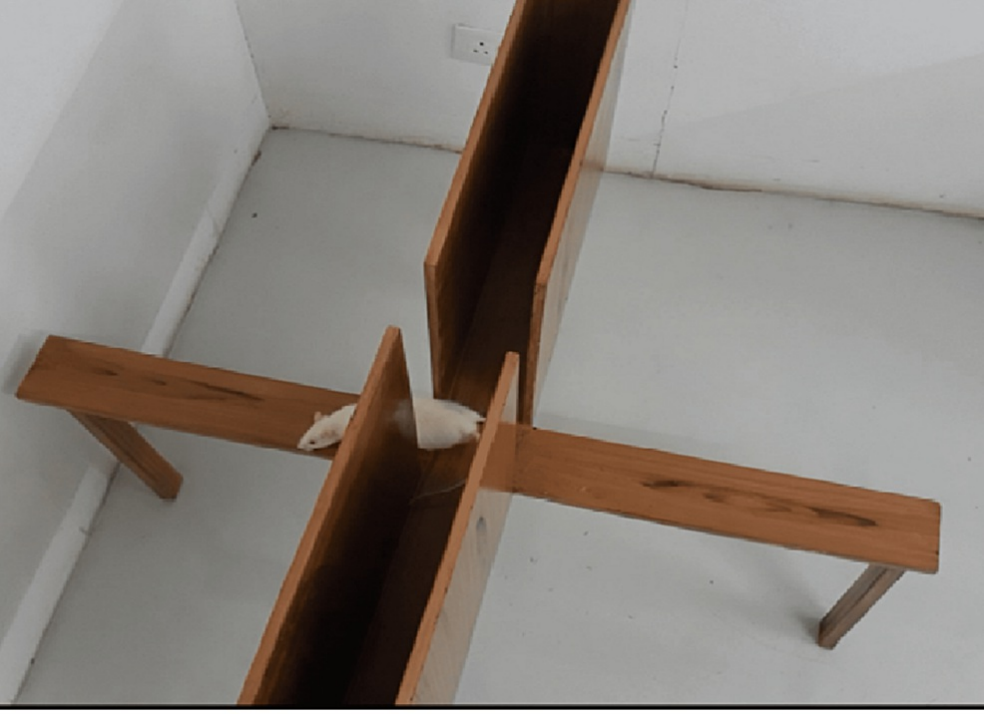
Rat exploring the open arm of the elevated plus maze

Figure 1 illustrates the elevated plus maze apparatus for the evaluation of anxiety in rodents. The mean time spent in the open arm by rats in the ramelteon group (0.25 mg/kg, 0.5 mg/kg, and 1 mg/kg) was not significantly higher as compared to the control group (Table 1).

**Table 1:** Effect of ramelteon on time spent in the open arm in the EPM paradigm in the acute study All values have been expressed as mean ± SEM. P value – one-way ANOVA followed by a 2-tailed Dunnet’s test (all the groups compared to control – vehicle). EPM: elevated plus maze; ANOVA: analysis of variance

S.No	Treatment (Dose: mg/kg)	Time spent in the open arm (mean)	Standard error of mean	P value*
Vehicle (DMSO)	-	19.67	1.333	-
Diazepam	1	84.67	32.220	0.003
Diazepam	0.5	22.00	2.206	1.000
Ramelteon	0.25	18.17	1.579	1.000
Ramelteon	0.5	26.50	2.513	1.000
Ramelteon	1	36.50	16.755	0.941
Ramelteon + Diazepam	0.25+0.5	32.50	3.063	0.990
Ramelteon + Diazepam	0.5+0.5	33.50	6.076	0.983
Ramelteon + Diazepam	1+0.5	37.83	4.045	0.909
Ramelteon + Flumazenil	1 + 1	39.50	16.468	0.859
Ramelteon + Picrotoxin	1 + 1	17.50	5.590	1.000
Ramelteon + Luzindole	1 + 2.5	20.33	6.566	1.000

**Figure 2:**
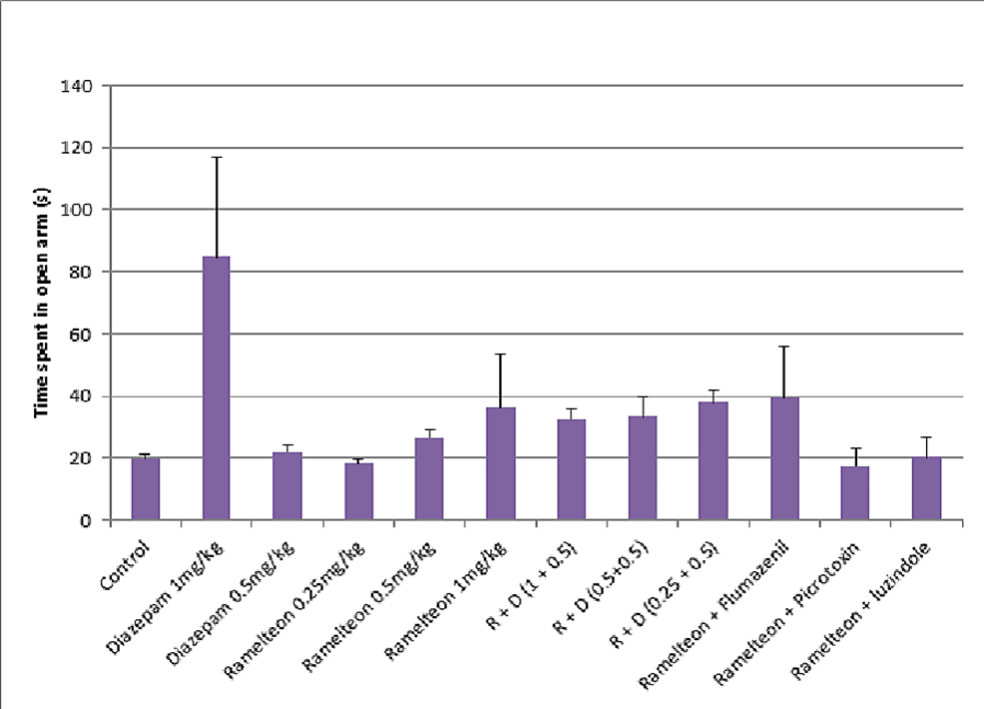
Bar diagram showing the mean time spent in the open arm across different groups in the acute study R: ramelteon group, D: diazepam group, R+D: ramelteon plus diazepam group

Figure 2 illustrates the effect of ramelteon in the EPM test in the acute study. There was no dose-dependent effect in the mean time spent in the open arm with different doses of ramelteon. A combination of ramelteon with antagonists (flumazenil, picrotoxin, and luzindole) produced no changes in the time spent in the open arm compared to ramelteon 1 mg.

Chronic Study

**Figure 3:**
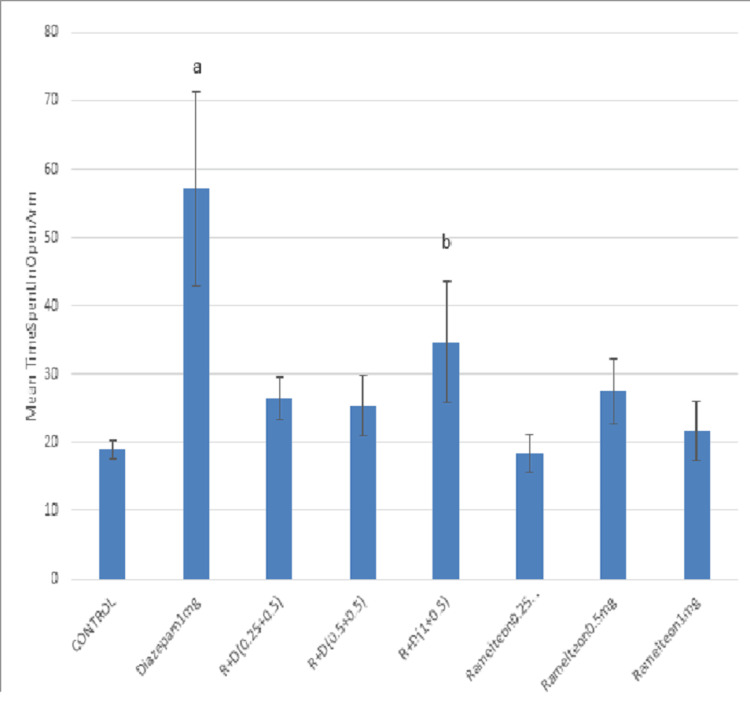
Bar diagram showing the mean time spent in the open arm across different groups in the chronic study R: ramelteon group, D: diazepam group, R+D: ramelteon plus diazepam group

In the chronic study, ramelteon 0.25, 0.5, and 1 mg/kg and a combination of these doses with diazepam 0.5 mg were administered for 14 days (Figure 3).

The mean time spent in the open arm in the combination group (1 mg/kg + 0.5 mg/kg) was significantly higher compared to the control group (Table 2).

**Table 2:** Effect of ramelteon on time spent in the open arm in the chronic study All values have been expressed as mean ± SEM. P value – one-way ANOVA followed by a 2-tailed Dunnet’s test (all the groups compared to control – vehicle) ANOVA: analysis of variance

S.No	Treatment (Dose: mg/kg)	Time spent in the open arm (mean)	Standard error of mean	P value*
Vehicle (DMSO)	-	19.00	0.683	
Diazepam	1	57.17	7.120	0.000
Ramelteon	0.25	18.50	1.384	1.000
Ramelteon	0.5	27.50	2.405	0.337
Ramelteon	1	21.67	2.171	0.991
Ramelteon + Diazepam	0.25+0.5	26.50	1.565	0.465
Ramelteon + Diazepam	0.5+0.5	25.33	2.216	0.636
Ramelteon + Diazepam	1+0.5	34.67	4.447	0.012

Hole board test

Acute Study

**Figure 4:**
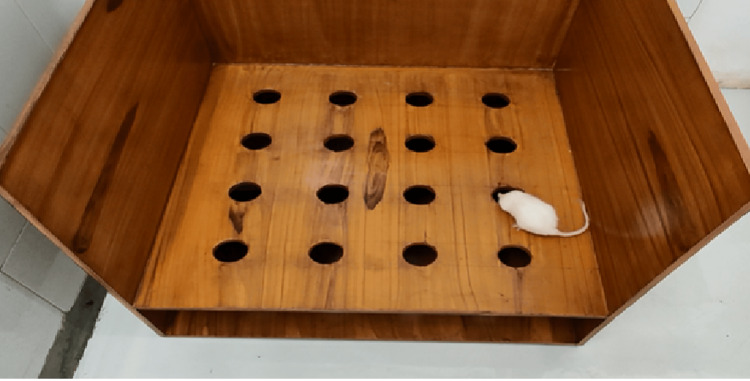
Head dipping behavior of rats in the hole board apparatus

The mean number of head dipping by rats in the ramelteon group (0.25 mg/kg, 0.5 mg/kg, and 1 mg/kg) was not significantly higher as compared to the control group (Figure 4).

**Figure 5:**
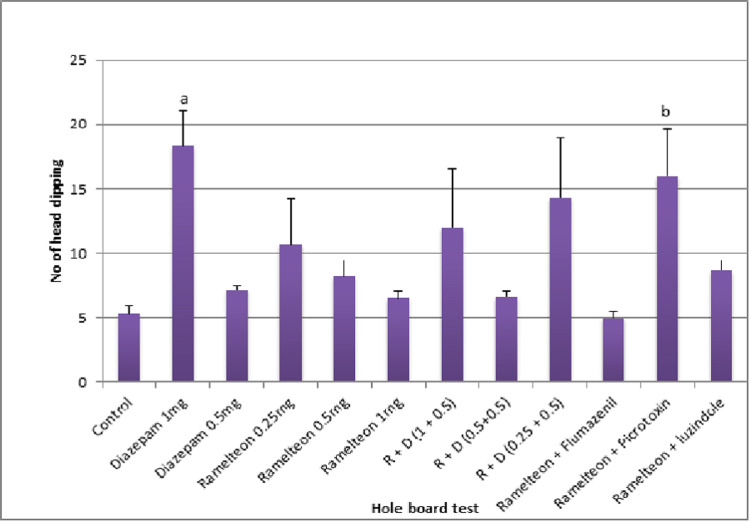
Bar graph showing the mean number of head dipping across different groups in the acute study

There was no dose-dependent effect in the mean number of head dippings with different doses of ramelteon. A combination of ramelteon with picrotoxin produced a significant increase in the mean number of head dippings as compared to the ramelteon 1 mg/kg group (Figure 5).

Chronic Study

**Figure 6:**
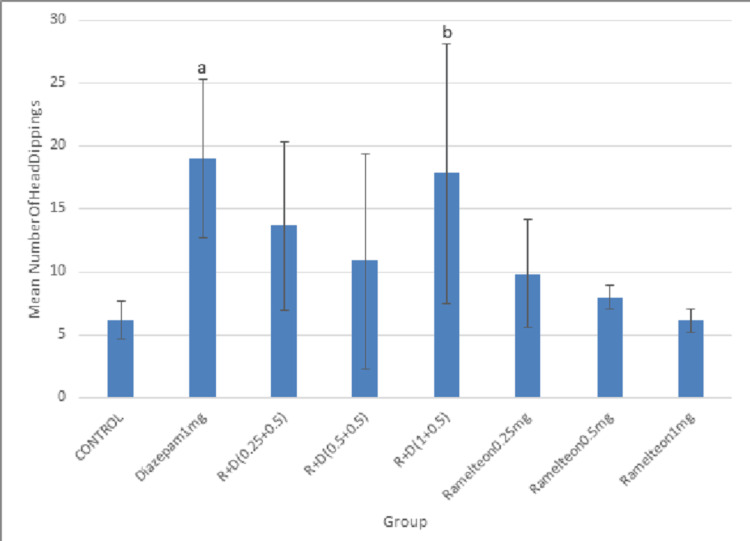
Bar diagram showing the mean number of head dippings across different groups in the chronic study

In the chronic study, ramelteon 0.25, 0.5, and 1 mg/kg and a combination of these doses with diazepam 0.5 mg were administered for 14 days. The mean number of head dippings in the combination group (1 mg/kg + 0.5 mg/kg) was significantly higher as compared to the control group (Figure 6).

Light-dark box test

Acute Study

**Figure 7:**
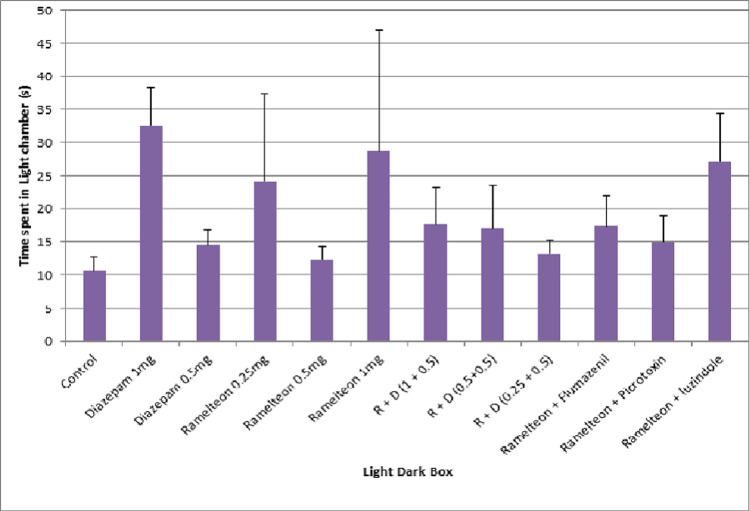
Bar diagram showing the mean time spent in the light chamber in the LDB test across different groups in the acute study LDB: light-dark box

The mean time spent in the light chamber by ramelteon group rats (0.25 mg/kg, 0.5 mg/kg, and 1 mg/kg) was not significantly higher as compared to the control group. Figure 7 illustrates the effect of ramelteon in the light-dark box in the acute study. There was no dose-dependent effect in the mean time spent in the light chamber with different doses of ramelteon. A combination of ramelteon with antagonists (flumazenil, picrotoxin, and luzindole) produced no changes in the time spent in the light chamber compared to the ramelteon 1 mg group. 

Chronic Study

**Figure 8:**
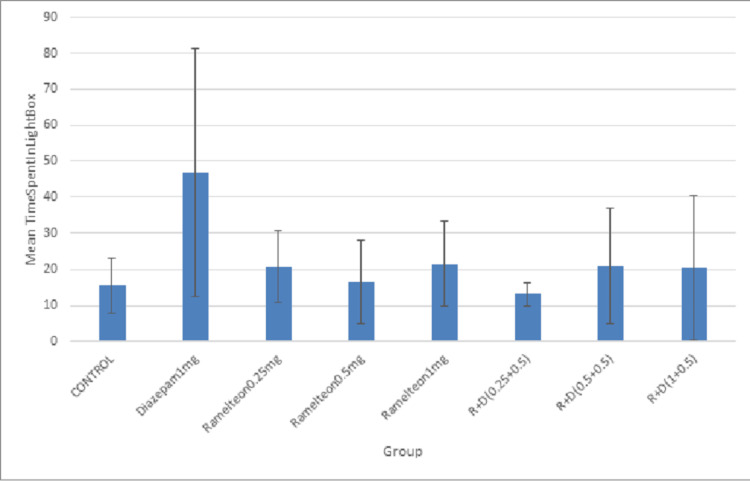
Bar diagram showing the mean time spent in the light chamber of the LDB test across different groups in the chronic study LDB: light-dark box

In the chronic study, ramelteon 0.25, 0.5, and 1 mg/kg and a combination of these doses with diazepam 0.5 mg/kg were administered for 14 days. The mean time spent in the light chamber in the diazepam 1 mg/kg group was significantly higher compared to the control group. Figure 8 illustrates the effect of ramelteon in the light-dark box in the chronic study.

Open field test

Acute Study

**Figure 9:**
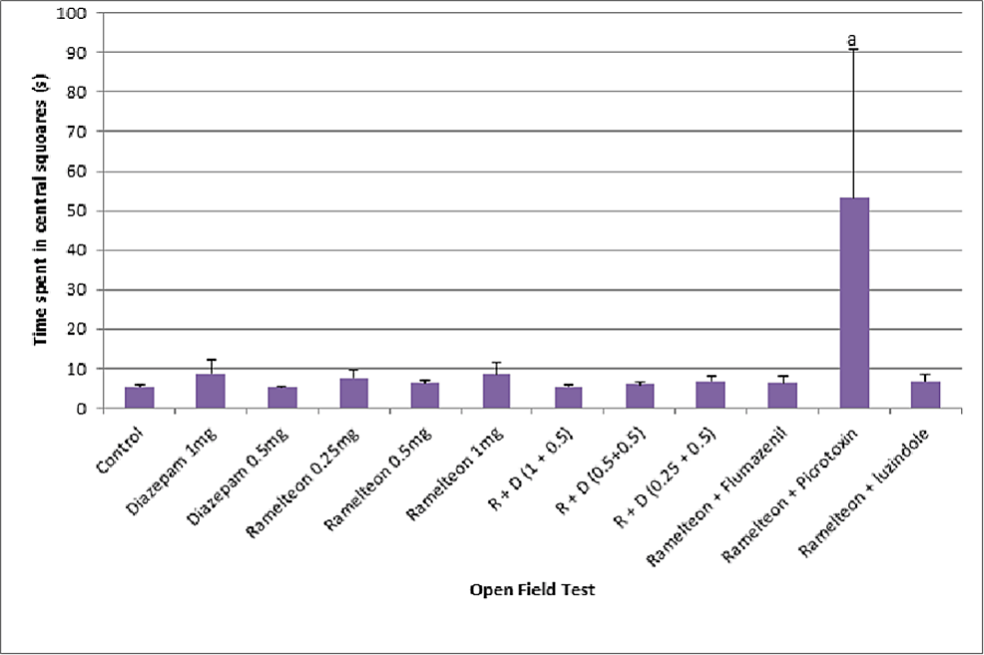
Bar diagram showing the mean time spent in central squares in the open field test across different groups in the acute study

The mean time spent in central squares by ramelteon group rats (0.25 mg/kg, 0.5 mg/kg, and 1 mg/kg) was not significantly higher as compared to the control group. Figure 9 illustrates the effect of ramelteon in the open field test in the acute study. There was no dose-dependent effect in the mean time spent in central squares with different doses of ramelteon. A combination of ramelteon with antagonist picrotoxin produced a significant increase in the time spent in the central squares as compared to ramelteon 1 mg/kg.

Chronic Study

**Figure 10:**
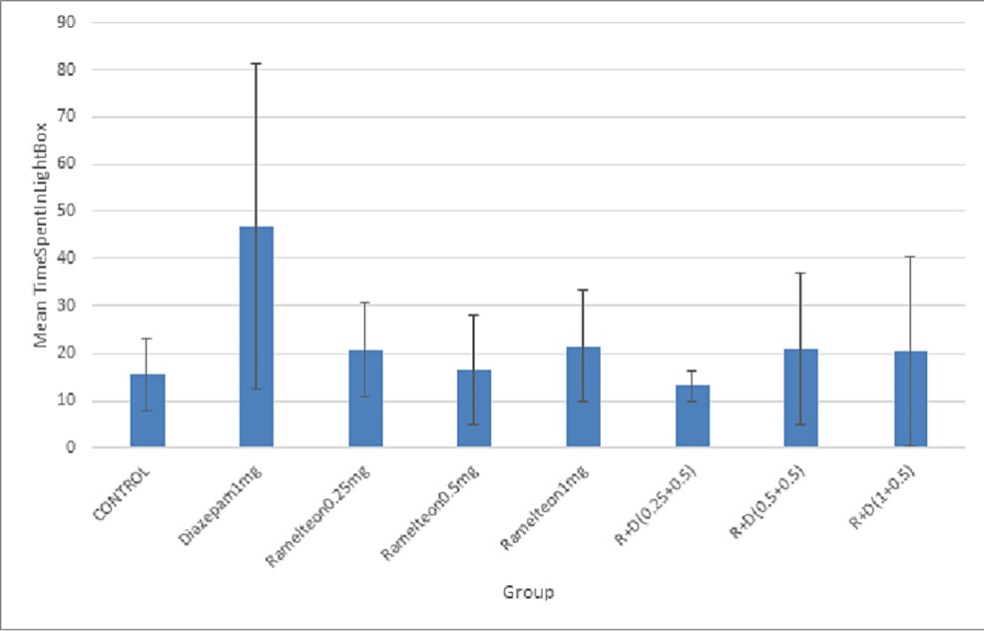
Bar diagram showing the mean time spent in central squares of the open field test across different groups in the chronic study

In the chronic study, ramelteon 0.25, 0.5, and 1 mg/kg and a combination of these doses with diazepam 0.5 mg/kg were administered for 14 days. The mean time spent in the central squares in ramelteon groups did not show significant changes. Figure 10 illustrates the effect of ramelteon in the open field test in the chronic study.

## Discussion

Melatonin’s sedative effects are mediated through the melatonin receptors MT1 and MT2. These receptors are present in the suprachiasmatic nucleus, cortex, thalamus, cerebellar cortex, and hippocampus. The MT1 and MT2 receptors present in the suprachiasmatic nucleus are responsible for the regulation of sleep. In humans and diurnal animals, melatonin acting on these receptors in the suprachiasmatic nucleus promotes sleep initiation and maintenance. While serum melatonin peaks at night in the majority of humans, it is found to be lower in aged people with insomnia. There is also inter-individual variation in serum melatonin levels, which is responsible for differences in peak alertness and circadian rhythm regulating the sleep-wake pattern. Various studies evaluated the use of melatonin in insomnia with sleep onset difficulty, insomnia in elderly people over 55 years, insomnia in children with neurodevelopmental disorders like autism spectrum disorder, circadian rhythm disorders like delayed sleep phase syndrome, jet lag and shift work disorder, and non-24 sleep-wake disorder, and successfully established its beneficial effect in the same [22,23]. Other research studies have shown its highly beneficial effect in delayed sleep phase syndrome (which is often confused with sleep onset insomnia) more than other sleep disorders [24,25].

Ramelteon exerts its effect through action on melatonin receptors. Ramelteon is a selective agonist of melatonin receptors MT1 and MT2. While melatonin has an action on MT3 receptors also, ramelteon does not have any action on MT3 receptors. Anti-anxiety and antidepressant effects were observed with melatonin receptor agonists agomelatine and piromelatine (Neu-P11) in animal models [26,27]. Ramelteon has, in fact, a six-fold greater affinity for MT1 and MT2 receptors than melatonin. Therefore, we hypothesized ramelteon may have similar anti-anxiety effects and to the best of our knowledge, we could not find any study in the literature that has evaluated the same. Four validated models - EPM, HBT, LDB, and OFT - were used to evaluate the anxiolytic effects. Ramelteon as an individual drug in different doses did not show an anxiolytic effect in any of the four models in both the acute and the chronic study.

A previous study has found that melatonin administration in the evening time at a dose of 10-75 mg/kg enhanced open-arm exploration by rodents [26]. Another study has proved that intra-amygdalar administration of 100 μg/kg melatonin enhanced the time spent in the open arm [27]. When melatonin has such positive results in several animal and human studies, melatonin receptor agonist ramelteon was also expected to have the same results. But our study findings suggest that ramelteon did not show any anxiolytic benefit in the EPM.

Pierrefiche et al. showed that melatonin decreased the head-dipping behavior of rats in the hole board test [28]. In our study, ramelteon did not increase or decrease the number of head dippings. Pierrefiche et al. also suggested that melatonin increased the time spent by rats in the light chamber of LDB, indicating its anxiolytic potential [28]. However, ramelteon did not increase the time spent in the light chamber.

Contrary to previous research, our study findings suggest that MT3 receptors may also be involved in the anxiolytic action of melatonin although MT1 and MT2 are identified as primary players mediating anti-anxiety effects. MT3 receptors are present in various brain sites, eyes, liver, and kidneys and have reductase detoxification enzyme activity. Ramelteon’s lack of action on MT3 receptors could have led to its inability to produce an anxiolytic effect. MT3 receptors are researched very less when compared to MT1 and MT2 receptors. More studies examining the MT3 receptors are needed to arrive at better conclusions.

Recent research has suggested that the activation of G protein-coupled melatonin receptors MT1 and MT2 by agonist ligands rarely leads to the activation of all downhill signaling pathways. The concept of biased agonism hints to us that there could be certain agonists that activate only a subset of the signaling pathway and result in one type of receptor behavior while another agonist may act on the same receptor differently [29,30]. This could be one of the reasons why ramelteon did not have the same effect as melatonin. Recent research has also discovered the possibility of homodimerization and heterodimerization of melatonin receptors with the same class and other classes of receptors like serotonin receptors [29]. These mechanisms might also be responsible for the different outcomes between melatonin and ramelteon. Molecular pharmacology of melatonin receptors is being studied rigorously and more important answers may unveil in the future soon.

Ramelteon as an individual drug did not produce any anxiolytic effect in both acute and chronic studies. But ramelteon 1 mg/kg in combination with diazepam 0.5 mg/kg administered orally for 14 days in the chronic study produced an anxiolytic effect in EPM and HBT while it failed to show anti-anxiety effects in OFT and LDB. The synergistic effect of diazepam on GABA-A receptors and ramelteon on MT1 and MT2 receptors could have produced the anxiolytic action.

We also applied the Tukey post hoc test after one-way ANOVA to do an inter-group comparison of the anxiolytic effect between the R + D (1 + 0.5) group and diazepam 1 mg/kg and diazepam 0.5 mg/kg in acute and chronic studies. Although this particular combination group has weak anxiolytic action when compared to the vehicle in the acute study with EPM and HBT, the results were inferior when compared to the diazepam 1 mg/kg group and 0.5 mg/kg group (P value = 0.229, 0.999, and 0.694, 0.993, respectively). In the chronic study with EPM, the anxiolytic effect of the R + D (1 + 0.5) group was found to be inferior to that of the diazepam 1 mg/kg group (P value = 0.040). In the chronic study with HBT, the anxiolytic effect of the R + D (1 + 0.5) group was as effective as the diazepam 1 mg/kg group (P value = 1.000).

Limitations

The typical anxiety paradigms, such as OFT, EPM, and LDB, are heavily reliant on locomotor activity, which can lead to false-negative or false-positive results. Anxiety in humans is not only limited to locomotive behavior but also to various other aspects of human life. Furthermore, the experiments are sensitive to minor changes in factors (such as lighting and researcher) making them difficult to replicate both inside and across laboratories. Blinding was not done in our study while conducting experiments in various groups. These were some limitations of our study.

## Conclusions

Ramelteon as a standalone drug in different doses failed to show an anxiolytic effect in the four validated models of anxiety evaluation - elevated plus maze, hole board test, light-dark box, and open field test in both acute and chronic studies. However, a combination of ramelteon 1 mg/kg and diazepam 0.5 mg/kg when administered orally for 14 days in the chronic study showed an anxiolytic effect in the elevated plus maze and hole board test. There was no significant gender difference in anxiety-like behavior in rats when administered with the vehicle and various drugs throughout the study except in the elevated plus maze where the male rats were found to spend more time in the open arm, indicating less anxiety. Further studies can evaluate the use of a fixed-dose combination of ramelteon and already-approved anxiolytic drugs to reduce the dose of the latter.
